# A curious case of persistently relapsing hyperkalemia in an ESRD patient on maintenance hemodialysis following bioprosthetic aortic valve replacement – a potential case for the use of the new agent, patiromer, for hyperkalemia management

**DOI:** 10.15171/jrip.2017.06

**Published:** 2016-09-24

**Authors:** Macaulay Amechi Chukwukadibia Onuigbo, Nneoma Agbasi, Fidelis Oguejiofor, Charles Odenigbo

**Affiliations:** ^1^Mayo Clinic College of Medicine, Rochester, MN 55905, USA; ^2^Department of Nephrology, Mayo Clinic Health System, Eau Claire, WI 54702, USA; ^3^North East London NHS Foundation Trust, United Kingdom; ^4^Department of Medicine, Nnamdi Azikiwe Teaching Hospital, PMB 5025, Nnewi, Anambra State, Nigeria

**Keywords:** Aortic valve replacement (AVR), Arrhythmia, Hemodialysis, Hyperkalemia, Patiromer, Prosthetic valves

## Abstract

Hyperkalemia is not uncommon in patients with end-stage renal disease (ESRD) on maintenance hemodialysis, often related to dietary indiscretion, following the prolonged inter-dialytic weekend interval in patients on thrice weekly hemodialysis treatments, and sometimes the adverse effects of medications such as RAAS blocking agents. Moreover, hyperkalemia following extended cardiac surgery can result from the use of high-potassium containing cardioplegic solutions used during cardiopulmonary bypass. Nevertheless, different from the foregoing, in the nephrology literature, there have been very rare reports of potentially life-threatening hyperkalemia following cardiac valve replacement procedure. We recently encountered an unusual case of persistent relapsing hyperkalemia complicating aortic valve replacement (AVR) in a 53-year-old obese Caucasian male patient despite repeated daily intermittent hemodialysis treatments. Our case report is the first to clearly demonstrate the yo-yo recurrence of newly observed episodes of hyperkalemia reappearing despite repeated treatments.

**Implication for health policy/practice/research/medical education:**

Hyperkalemia following extended cardiac surgery can result from the use of high-potassium containing cardioplegic solutions used during cardiopulmonary bypass. Nevertheless, there is the rare syndrome of hyperkalemia following aortic valve replacement due to the mechanical destruction of formed elements in the blood. We herein present such a case that required repeated daily hemodialysis treatments for nearly a week before the hyperkalemia abated. We entertained the possible role of the oral potassium binder, Patiromer, in such a clinical scenario.

## Introduction

Hyperkalemia is a frequent finding in end-stage renal disease (ESRD) patients and the causes arise from different mechanisms such as increased intake, cellular shifts and reduced excretion. Often it arises from a combination of these. Commonly implicated causes of hyperkalemia in ESRD patients include high-potassium diet, metabolic acidosis, blood transfusions, diabetes mellitus, drugs (non-selective beta blockers, nonsteroidal anti-inflammatory drugs [NSAIDS], angiotensin receptor blockers, angiotensin converting enzyme inhibitors) and advanced stages of heart failure (1).

The introduction of valve replacement surgery has dramatically improved the outcome of patients with valvular heart disease with approximately 90000 valves implanted in the United States each year and 280000 worldwide (2).

It is generally acknowledged that prosthetic valves have suboptimal hemodynamics and can therefore be complicated by post-placement hemolysis (3,4). There have been few reports of post-valve replacement hyperkalemia (3,4). Nevertheless, life-threatening electrolyte disturbances caused by such hemolysis are very rare, even in dialysis patients (5). Recently, we encountered an unusual case of persistent relapsing potentially life-threatening hyperkalemia, first appearing only a few hours following minimally invasive aortic valve replacement (AVR), and that subsequently required repeated daily intermittent hemodialysis treatments for nearly a week before the hyperkalemia abated. We discuss here, previously reported mechanisms that lead to this syndrome and in addition posit a new previously unreported mechanism.

## Case Presentation

A 53-year-old obese Caucasian male smoker, on maintenance hemodialysis three times weekly for anuric ESRD for about five years, was admitted for surgical treatment of symptomatic severe aortic valve stenosis in January 2015. Presenting symptoms included intermittent spells of heaviness in his chest that would last a few minutes to an hour, dyspnea with walking short distances, and he was extremely limited in physical activity. The past medical history included hypertension, type II diabetes mellitus, dyslipidemia and a prior history of coronary artery stents placed in the circumflex coronary and right coronary arteries a few years back. In addition, he had needed a pericardiocentesis procedure on June 18, 2014 for pericardial effusion with tamponade resulting from uremic pericarditis in an outside hospital. Successively, a diagnostic coronary angiography in November 2014 in our hospital had demonstrated widely patent coronary artery stents. His outpatient medications were metoprolol, nifedipine, glipizide, minoxidil, sevelamer, baclofen, oxycodone, pravastatin, aspirin, darbopoetin, sertraline, eszopiclone, thiamine, loperamide, multivitamins, folic acid and iron tablets.

Preoperative examination was unremarkable - pulse 92 per minute and regular, blood pressure 124/72 mm Hg. electrocardiogram (ECG) was abnormal with normal sinus rhythm, left axis deviation, left bundle branch but no acute changes. Preoperative transesophageal echocardiogram demonstrated preserved left ventricular function with a critical aortic stenosis and mild aortic insufficiency, and the left ventricular ejection fraction was 75%. The patient consented and subsequently underwent a minimally invasive AVR procedure employing a 25 mm St. Jude Epic stented tissue valve in late January 2015, under cardioplegia, with a bypass time of 185 minutes and a cross clamp time of 122 minutes. He received 550 mL of autologous blood intraoperatively given as one liter of adenosine enriched warm blood. Post-operative transesophageal echo demonstrated left ventricular ejection fraction of 50% with a well seated and normal functioning aortic valve prosthesis. The heart resumed spontaneous rhythm after cardioplegia and he was AV paced to 90 beats/min without the need for inotropic support.

**Figure 1:**
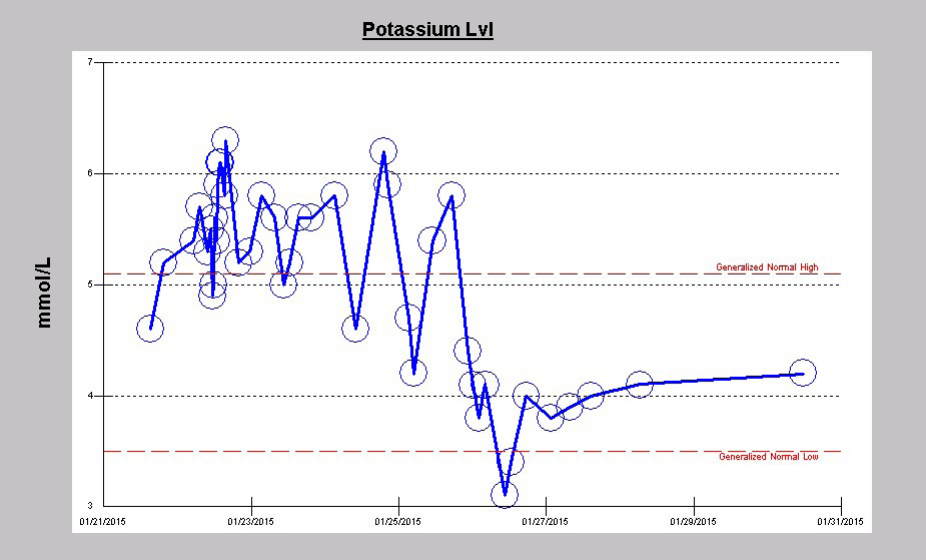
The serum potassium trajectory following minimally invasive AVR with persistently relapsing hyperkalemia for nearly a week despite repeated more than daily hemodialysis treatments.

Post-operatively, however, the patient exhibited progressively rising hyperkalemia. Vital signs were otherwise stable and he was extubated. Preoperative serum potassium was 4.6 mmol/L and post operatively had quickly risen to 6.3 mmol/L (Figure 1). An urgent repeat transthoracic echocardiogram that evening revealed normal left ventricular function with ejection fraction of 75%, a well seated aortic valve prosthesis, no perivalvular leak, and a normal aortic valve gradient by Doppler of only 6 mm Hg. An urgent 4-hour hemodialysis treatment was initiated using the continuous veno-venous hemodialysis (CVVHD) protocol with a NxStage machine, blood flow rate via a left upper extremity arteriovenous fistula of 300-400 mL/min, ultrafiltration target of 2-3 kg, CVVHD fluid rate of 4-4.8 L/h, and a filtration fraction of 25%-30%. The dialysate potassium concentration used was 2 mmol/L. Post-dialysis serum potassium concentration was 5.3 mmol/L. The next morning serum potassium had increased again to 5.8 mmol/L. He again had another 4-hour run of CVVHD, but this time using a dialysate bath potassium concentration of 3 mmol/L. The second day post-operative, he again needed another 4-hour CVVHD treatment for relapsing hyperkalemia with a dialysate bath potassium concentration of 2 mmol/L and the potassium fell from 5.8 mmol/L to 4.6 mmol/L (Figure 1). To our surprise, while still in the coronary care unit (CCU), 9 hours post-dialysis, still on the same post-operative day 2, his closely monitored serum potassium had risen again to 6.2 mmol/L, thus necessitating a second 4-hour CVVHD run with a dialysate potassium bath concentration of 2 mmol/L. This time, the serum potassium was reduced from 6.2 mmol/L to 4.2 mmol/L post-dialysis. The next day, post-operative day 3, serum potassium had increased again to 5.8 mmol/L and he therefore had an extended 6-hour CVVHD run, this time with a dialysate bath concentration of only 1.0 mmol/L (Figure 1). Serum potassium fell to 3.8 mmol/l following the 6-hour treatment. Subsequently, over the ensuing three days, although his morning serum potassium levels remained within the normal range, he still received scheduled intermittent daily 4-hour CVVHD treatments with dialysate potassium bath concentration of 2.0 mmol/L (Figure 1).

## Other laboratory evaluations

**Figure 2:**
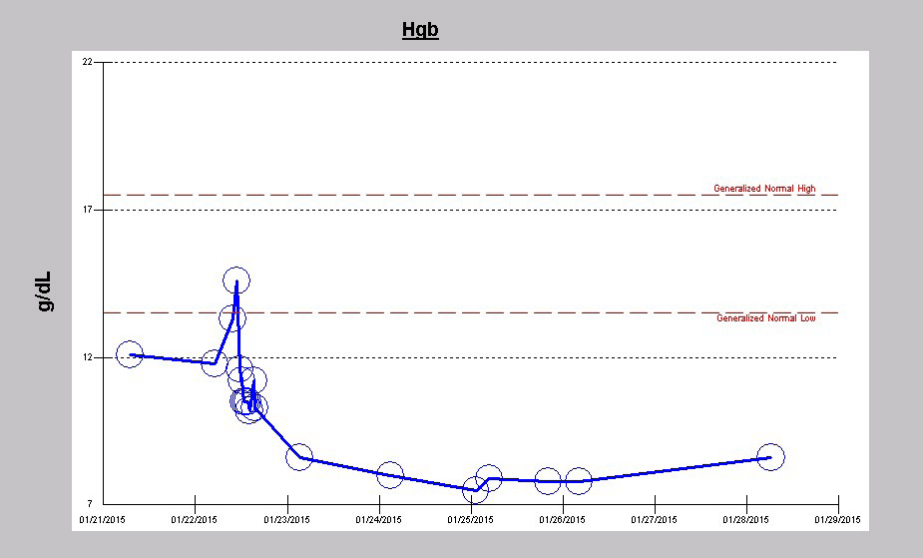
Translational changes in hemoglobin levels following minimally invasive AVR consistent with post-placement erythrocyte fragmentation with resulting hemolysis.

**Figure 3:**
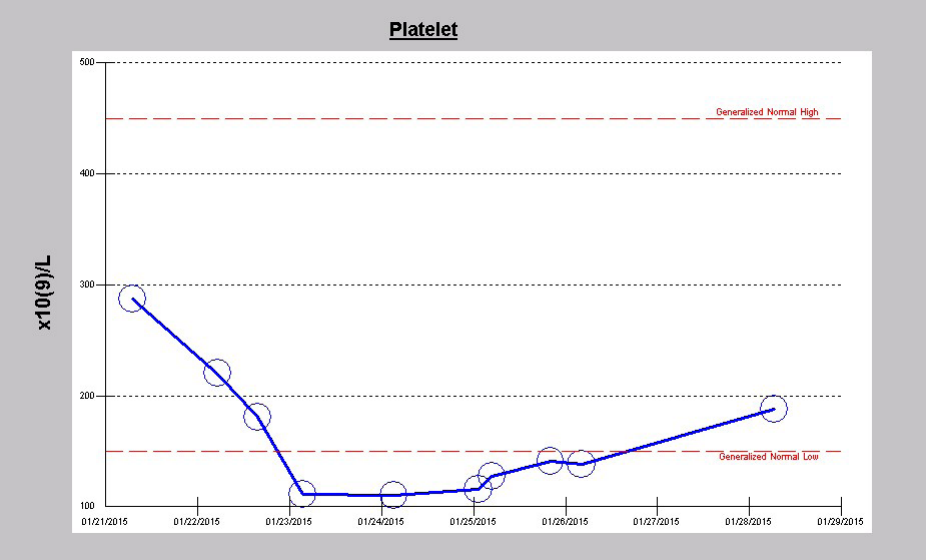
Translational changes in platelet count following minimally invasive AVR consistent with post-placement platelet breakdown and lysis.

**Figure 4:**
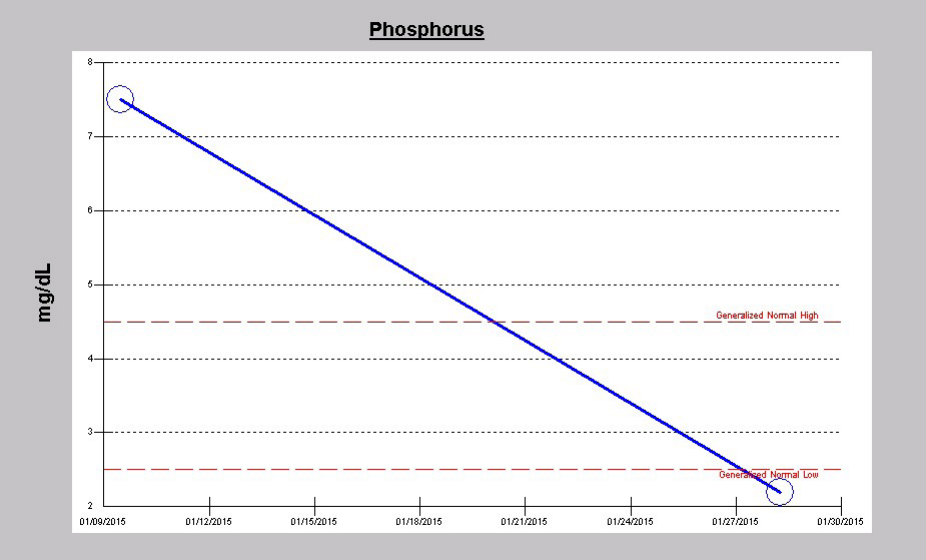
Translational changes in serum phosphorus in dialysis patient following minimally invasive AVR and repeated more than daily hemodialysis treatments.

His liver panel preoperatively including aspartate transaminase (AST), alanine transaminase (ALT) and total bilirubin were normal but were never repeated during the course of the hospitalization. On post-operative day 2, laboratory investigations revealed elevated lactate dehydrogenase (LDH) of 307 U/l (122-222 units/L). Blood for haptoglobin and peripheral smear examination for schizonticytes were not performed. Of note, his hemoglobin levels fell significantly following the surgery after the initial rise intraoperatively following intraoperative packed red blood cells transfusions (Figure 2). Furthermore, he showed an abrupt thrombocytopenia following surgery with platelet count decreasing from 287×10^3^/µL preoperative down to 111 ×10^3^/µL on post-operative day one. Platelet count subsequently improved and had normalized to 188 ×10^3^/µL on the day he was discharged (Figure 3). As a result of the repeated hemodialysis treatments, his usually high levels of phosphorus was now in the hypophosphatemic range at discharge (Figure 4).

He was subsequently discharged 6 days after the AVR, with stable normal levels of serum potassium, on his previous outpatient medications together with warfarin anticoagulation which cardiology recommended to be administered for a period of two months for the Epic stented tissue valve replacement.

## Discussion

Hyperkalemia is a potentially serious condition that can result in life-threatening cardiac arrhythmias and is associated with an increased mortality risk (6). Our patient, following minimally invasive AVR for symptomatic critically severe calcific aortic valve stenosis (55 mm gradient) had developed rapidly occurring and persistently relapsing potentially life-threatening hyperkalemia requiring a total of eight 4-6 hour intermittent hemodialysis treatments using low potassium concentration dialysate baths. To our knowledge, this is the first such reported case exhibiting such relapsing persistence of hyperkalemia over several days, and that, despite repeated intermittent hemodialysis interventions. Notably there was no potentially triggering supraventricular arrhythmia, and furthermore there was clear echocardiograhic evidence of a well seated and normally functioning Epic stented tissue valve without any perivalvular leak or abnormality.

Prosthetic heart valves have suboptimal hemodynamics and can therefore be complicated by post-placement hemolysis; there have been few reports of post-valve replacement hyperkalemia (3,4). During the past 40 years, approximately 3 million prosthetic heart valves have been implanted worldwide (5). Hemolysis more often complicates a mechanical valve more frequently than bioprosthetics as shown in a prospective study of 172 patients with mechanical valves and 106 with bioprosthetics (4). Hemolysis complicated 26% of the patients with mechanical valves and 5% of those with bioprosthetics (4). Rose et al described hemolytic anaemia after heart valve replacement as far back as 1954 (7). Skoularigis et al reported that in 80 patients with Medtronic Hall (MH) prostheses and normal mechanical function hemolysis was never severe (8). The mechanisms of hemolysis in patients with artificial valves include high shear stress on the artificial surface of the prosthetic valve, even in normally functioning valves (3,4). According to Mecozzi et al, hemolysis was evaluated in 172 patients with a mechanical prosthesis (53 CarboMedics and 119 Sorin Bicarbon) and in 106 patients with a bioprosthesis (15 St. Jude Medical Toronto [SJM], 19 Baxter Perimount, and 72 Medtronic Hall [MH]) in the aortic position, mitral position, or both (4). Although none of the 278 patients experienced decompensated anemia, however at 12 months, mild subclinical hemolysis was identified in 49 patients, 44 (26%) with a mechanical prosthesis and 5 (5%) with a bioprosthesis (*P*<0.001) (4). In an earlier analysis, Skoularigis et al evaluated 170 patients with SJM and 80 patients with MH prostheses, with normal mechanical function (8). The presence and severity of hemolysis was assessed on the basis of serum lactic dehydrogenase, serum haptoglobin, blood hemoglobin and reticulocyte levels as well as the presence of schistocytes (8). Overall, patients with SJM prostheses had greater frequency (51.2 vs 18.7%, *P*<0.005) and severity (*P*<0.005) of hemolysis than patients with MH prostheses, irrespective of position and size (8).

However, life-threatening electrolyte disturbances caused by hemolysis are very rare, even in dialysis patients (5). In the 2007 report by Papadogiannakis et al, of life-threatening hyperkalemia in an ESRD patient occurring several years after prosthetic AVR, the precipitating factor was a symptomatic re-entrant tachycardia of 180 beats per minute that subsequently responded to two intravenous 5 mg doses of verapamil, given 10 minutes apart, with return of normal sinus rhythm (5). Transient shear stress induced hemolysis was precipitated by tachycardia-induced turbulence over the 3-year old MH aortic valve prosthesis for severe rheumatic heart disease (5).

Besides, literature review shows that hemolysis is uncommon with bioprosthetics. Its incidence is further reduced by use of stentless bioprosthetics (9). Our patient had a tissue stented bioprosthesis. Hyperkalemia resulting from the fragmentation hemolysis is attributed to hemodynamic turbulence such that red blood cells can be damaged by the turbulent flow fields associated with current prosthetic mechanical heart valve designs (10). The mechanisms of hemolysis in patients with artificial valves include high shear stress on the artificial surface of the prosthetic valve, even in normally functioning valves (3,4). Prosthetic valves have suboptimal hemodynamics; mechanical valves require anticoagulation and tissue valves wear out over time; moreover serious complications of prosthetic valves occur at a rate of about 2% to 3% per patient-year and such complications include thromboembolism, prosthesis-patient mismatch, structural valve dysfunction, endocarditis, and hemolysis (3). Perivalvular leak or periprosthetic valvular regurgitation is another identified pathogenetic factor associated with hemolysis in patients with prosthetic valves (3,11). This was absent in our patient presented in this report.

Rose et al as far back as 1954 described significant drops in hematocrit after prosthetic aortic heart valve replacement (7). In our patient the acute drop in hemoglobin following the valve replacement operation is consistent with this observation of intravascular fragmentation of the red blood cells (Figure 2). In addition, our patient demonstrated an acute drop in platelet count immediately following the valve operation (Figure 3). We posit, that an additional previously unreported causative factor in post-prosthesis hyperkalemia is platelet fragmentation (12,13).

## Epilogue on hyperkalemia – a word on patiromer

Although there are effective therapeutic options for the short-term acute management of hyperkalemia, the available strategies for chronic control of high potassium levels have limited effectiveness (6). As such, there is an important unmet need for novel therapeutic options for the chronic management of patients at risk for hyperkalemia. Potential therapies in development may change the treatment landscape in the near future (6,14,15).

As recalled by Paton in a recent review article, the U.S. Food and Drug Administration (FDA) approved Patiromer to treat hyperkalemia on October 21, 2015, making it the first agent approved for this condition in 50 years (16). Patiromer was developed by Relypsa, Inc (14-16). The active ingredient is Patiromer sorbitex calcium which consists of the active moiety, Patiromer, a nonabsorbed potassium-binding polymer, and a calcium-sorbitol counterion. In the colon, Patiromer exchanges calcium for potassium thus causing a fall in serum potassium. Trials have shown that Patiromer reduces serum potassium in patients with mild, moderate and moderate to severe hyperkalemia to the normal range. It has also been used successfully in patients with chronic kidney disease and/or heart failure. It has also allowed the use of the mineralocorticoid antagonist spironolactone at full dosage in patients with chronic kidney disease and/or heart failure who were already receiving a renin-angiotensin-aldosterone system inhibitor. Adverse effects have mostly been gastrointestinal in nature and have not caused patients to discontinue treatment in unacceptable numbers. Of note, we must acknowledge here that Patiromer is not specifically approved for use in ESRD patients on hemodialysis. Arguably, if such post-AVR hyperkalemia persisted in a non-ESRD patient, there could indeed be a place for the chronic use of Patiromer to mitigate chronic hyperkalemia. Usually, though, such episodes of post valve replacement hyperkalemia are short-lived.

## Conclusion

Finally, we note that post-AVR hyperkalemia represents the impact of the suboptimal hemodynamics of prosthetic valves, mechanical valves more so than bioprosthetics, with the resultant turbulence and high shear stress on the artificial surface of the prosthetic valve leading to fragmentation of both erythrocytes and platelets, a process that may last for several days until the prosthetic valve is coated with newly formed endothelial cells (3,4,7,8). Previously only erythrocyte breakdown had been described (3,4,7,8). Besides, our case presentation has implicated platelets breakdown as an additional causative factor in post-AVR hyperkalemia. Although rare, since this can be potentially life-threatening, the medical team must remain vigilant for such relapsing occurrences following prosthetic AVR in the immediate post-operative period.

## Authors’ contribution

MACO; conception, design, acquisition of data, data analysis, interpretation of data, literature review, drafting the article and final approval of manuscript. MAR; critical revising for important intellectual content, design, final approval of manuscript. FO; literature review, drafting the article and final approval of manuscript. CO; literature review, drafting the article and final approval of manuscript.

## Conflicts of interest

The authors report no conflicts of interest. The authors alone are responsible for the content and writing of the article.

## Ethical considerations

Ethical issues (including plagiarism, data fabrication, double publication) have been completely observed by the authors. Written consent was obtained from the patient for publication of the study.

## Funding/Support

None.
